# DdrA, DdrD, and PprA: Components of UV and Mitomycin C Resistance in *Deinococcus radiodurans* R1

**DOI:** 10.1371/journal.pone.0069007

**Published:** 2013-07-01

**Authors:** Kathiresan Selvam, Jana R. Duncan, Masashi Tanaka, John R. Battista

**Affiliations:** 1 Department of Biological Sciences, Louisiana State University and A & M College, Baton Rouge, Louisiana, United States of America; 2 Department of Molecular Immunology and Inflammation, National Center for Global Health and Medicine, Tokyo, Japan; University of Massachusetts Medical School, United States of America

## Abstract

Mutants created by deleting the *ddrA*, *ddrB*, *ddrC*, *ddrD*, and *pprA* loci of *Deinococcus radiodurans* R1alone and in all possible combinations of pairs revealed that the encoded gene products contribute to this species’ resistance to UV light and/or mitomycin C. Deleting *pprA* from an otherwise wild type cell sensitizes the resulting strain to UV irradiation, reducing viability by as much as eight fold relative to R1. If this deletion is introduced into a *ΔddrA* or *ΔddrD* background, the resulting strains become profoundly sensitive to the lethal effects of UV light. At a fluence of 1000 Jm^-2^, the *ΔddrA ΔpprA* and *ΔddrD ΔpprA* strains are 100- and 1000-fold more sensitive to UV relative to the strain that has only lost *pprA*. Deletion of *ddrA* results in a 100 fold increase in strain sensitivity to mitomycin C, but in backgrounds that combine a deletion of *ddrA* with deletions of either *ddrC* or *ddrD*, mitomycin resistance is restored to wild type levels. Inactivation of *ddrB* also increases *D. radiodurans* sensitivity to mitomycin, but unlike the *ddrA* mutant deleting *ddrC* or *ddrD* from a *ΔddrB* background further increases that sensitivity. Despite the effect that loss of these gene products has on DNA damage resistance, none appear to directly affect either excision repair or homologous recombination suggesting that they participate in novel processes that facilitate tolerance to UV light and interstrand crosslinks in this species.

## Introduction

*Deinococcus radiodurans* R1 is the type species for the *Deinococcaceae*, a family of bacteria [1,2] exhibiting extraordinary resistant to the lethal effects of many DNA damaging agents, including ionizing radiation (IR), ultraviolet (UV) light, and interstrand cross-linking agents (ICLs) [3]. For a vegetative cell, *D. radiodurans* R1 is particularly resistant to UV radiation, surviving doses as high as 750 Jm^-2^ with minimal loss of viability [4]. In comparison, the D_37_ dose (the dose that on average is necessary to inactivate a cell) for *E. coli* B/r is approximately 30 Jm^-2^ [5]. UV exposures as high as those that *D. radiodurans* tolerates introduce enormous amounts of DNA damage. Exposure to a fluence of 500 Jm^-2^ UV should generate 5000 thymine-containing pyrimidine dimers per genome in this species [6,7], an average of one lesion of this type for every 640 base pairs. Clearly, *D. radiodurans* expresses efficient mechanisms for dealing with UV-induced DNA damage, and it is not unreasonable to assume that UV resistance in this species might rely on processes not found in more UV sensitive microorganisms.

*D. radiodurans* R1 encodes all of the components of the UvrABC-dependent nucleotide excision repair (NER) system characterized in many species including *E. coli* [8]. In contrast to *E. coli*, inactivating NER does not reduce *D. radiodurans* resistance to UV light. NER-defective strains of *D. radiodurans* only become sensitive to UV when a second locus, designated *uvs* (DR1819) [8], is also inactivated [9,10]. The *uvs* gene encodes a UV DNA damage endonuclease similar to that reported in *Schizosacchromyces* *pombe* [11,12] that is capable of completely compensating for the loss of NER. Likewise, if the *uvs* gene is inactivated in an otherwise wild type background, the resulting strain exhibits near wild-type levels of UV resistance, because of the overlapping action of NER. Thus, while the presence of two excision repair systems is a distinctive characteristic of *D. radiodurans*, the cell does not require this redundancy for UV resistance, making it difficult to argue that the presence of two repair systems contributes significantly to the species’ extraordinary tolerance of UV light. Mutational inactivation of the *polA* locus of R1 results in strains that are sensitive to all forms of DNA damage [13,14], indicating that DNA polymerase I of *D. radiodurans* (like its counterpart in *E. coli*) plays a central role in repair of UV-induced damage, presumably through gap filling after excision repair or in strand extension following recombinational repair. Deletion of *recA* also results in a strain of *D. radiodurans* that is extremely sensitive to DNA damage [15,16] presumably due to the cell’s inability to carry out homologous recombination.

From the above discussion, it seems apparent that although *D. radiodurans* expresses several proteins analogous to those known to counteract the lethal effects of UV light [9] in other species, there is nothing about these proteins that suggests an explanation for the extraordinary UV resistance of *D. radiodurans*.

Five novel gene products have been linked to the IR resistance of *D. radiodurans* R1 [16]. The transcripts of these genes – designated *ddrA* (DR0423), *ddrB* (DR0070), *ddrC* (DR0003), *ddrD* (DR0326), and *pprA* (DRA0346) – are the five most highly induced in response to IR and desiccation (a stress that like IR induces DNA double strand breaks). Mutants created by deleting these five loci alone and in all possible pairs revealed that each gene product partially contributed to IR resistance, but the functions of these proteins has remained obscure.

In the present study, the same collection of mutants is analyzed to determine if these gene products also affect *D. radiodurans*’ capacity to survive exposure to UV light and mitomycin C (MC). We report that the PprA, DdrA, and DdrD gene products are components of overlapping processes that make a major contribution to UV resistance. Deletion of PprA from R1 sensitizes R1 to UV light, whereas inactivation of DdrA and DdrD in a *pprA* ^+^ background does not alter UV resistance. When deletions of *ddrA* or *ddrD* are combined with a *pprA* deletion, cultures are as much as 1000-fold more sensitive to the lethal effects of UV light relative to their wild type parent. The functions of these proteins do not appear related to either excision repair or homologous recombination, suggesting they mediate previously undefined mechanisms that facilitate tolerance of UV-induced DNA damage in *D. radiodurans*.

## Materials and Methods

All strains used in this study are described in Table 1. The *D. radiodurans* strains are derived from the R1 type strain (ATCC13939), and were grown at 30^o^C in TGY broth (0.8% tryptone, 0.1% glucose, 0.4% yeast extract) or on TGY agar (1.5% agar) as described previously [17,18]. *D. radiodurans* survival following exposure to UV radiation and mitomycin C was assessed in cultures during exponential phase growth (at a density between 2 x 10^6^ and 4.5 x 10^7^ CFU/ml). Prior to exposure to UV light, cells were harvested by centrifugation and re-suspended in an equivalent volume of sterile saline (0.9% NaCl). One ml aliquots were placed in a sterile Petri dish and irradiated uncovered at 25°C using a germicidal lamp emitting at a calibrated dose rate of 25 Jm^-2^s^-1^ UV–C. Irradiated cultures were diluted in saline, prior to plating on TGY agar. Cultures to be exposed mitomycin C were grown to the appropriate density, harvested by centrifugation, and re-suspended in an equivalent volume of TGY broth containing 20 µg/ml mitomycin C. Cultures were incubated at 30^o^C and aliquots of the MC-treated culture were removed at ten minute intervals, washed twice in an equal volume of TGY broth, diluted, and plated on TGY agar. With the exception of TNK113 *ΔddrC ΔddrD*, survival was scored using colony counts three days after plating. TNK113 is very slow growing and colony formation was not evident until seven days after plating.

**Table 1:** Strain List.

Strain	Description	Reference
LSU2000	As R1 but *uvrA1*, *uvs*::Tn*DrCat*	[9]
TNK101	As R1 but *ΔddrC*::p*kat*-*aadA*	[16]
TNK102	As R1 but *ΔddrB*::p*kat*-*cat*	[16]
TNK103	As R1 but *ΔddrD*::p*kat*-*kan*	[16]
TNK104	As R1 but *ΔddrA*::p*kat*-*hyg*	[16]
TNK105	As R1 but *ΔpprA*::p*kat*-*aadA*	[16]
TNK106	As R1 but *ΔrecA*::p*kat*-*cat*	[16]
TNK112	*ΔddrB*::p*kat*-*cat*, *ΔddrC*::p*kat*-*aadA*	[16]
TNK113	*ΔddrC*::p*kat*-*aadA*, *ΔddrD*::p*kat*-*kan*	[16]
TNK114	*ΔddrA*::p*kat*-*hyg*, *ΔddrC*::p*kat*-*aadA*	[16]
TNK115	*ΔddrC*::p*kat*-*aadA*, *ΔpprA*::p*kat*-*aadA*	[16]
TNK116	*ΔddrB*::p*kat*-*cat*, *ΔddrD*::p*kat*-*kan*	[16]
TNK117	*ΔddrA*::p*kat*-*hyg*, *ΔddrB*::p*kat*-*cat*	[16]
TNK118	*ΔddrB*::p*kat*-*cat*, *ΔpprA*::p*kat*-*aadA*	[16]
TNK119	*ΔddrA*::p*kat*-*hyg*, *ΔddrD*::p*kat*-*kan*	[16]
TNK120	*ΔddrD*::p*kat*-*kan*, *ΔpprA*::p*kat*-*aadA*	[16]
TNK121	*ΔddrA*::p*kat*-*hyg*, *ΔpprA*::p*kat*-*aadA*	[16]

All strains are derived from *Deinococcus radiodurans* R1 ATCC13939.

## Results

### Deletion of *pprA* (DRA0346) sensitizes *D. radiodurans* R1 to UV light

**Figure 1:**
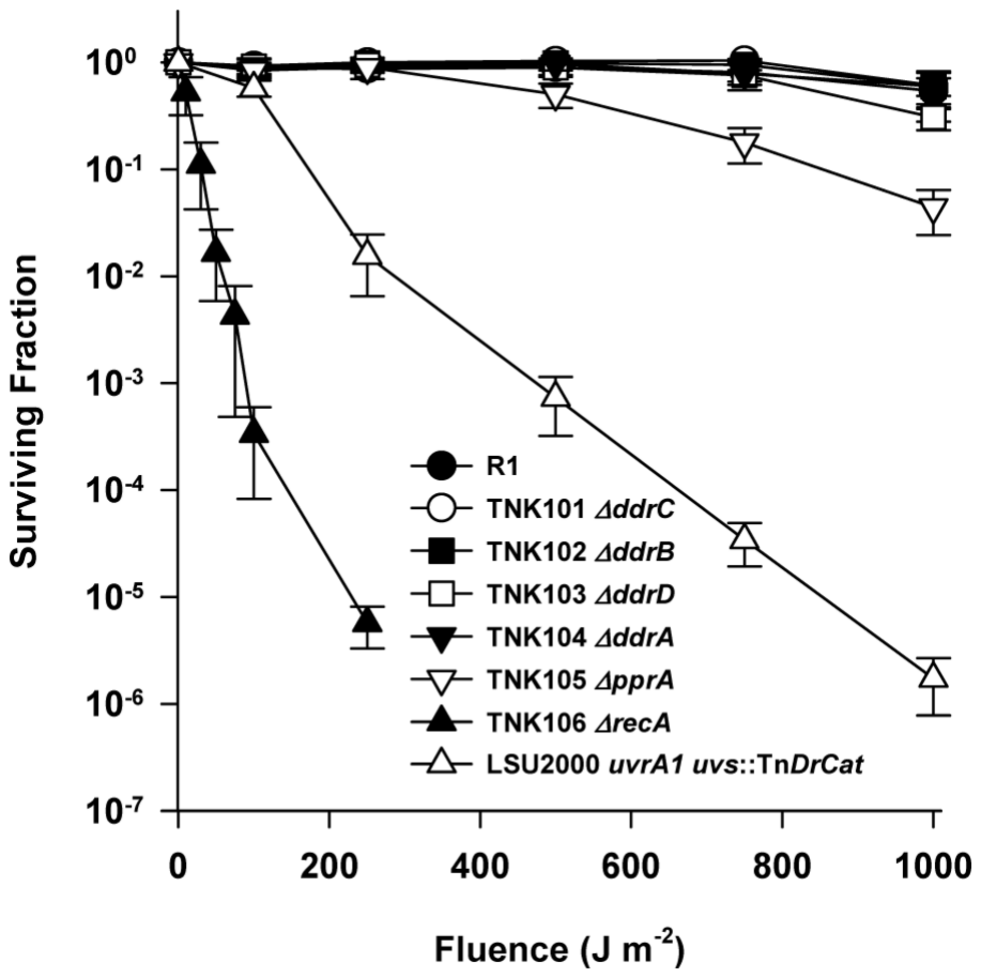
Survival curves for *ΔddrA*, *ΔddrB* *, ΔddrC*, *ΔddrD*, *and* *ΔpprA* derivatives of *D. radiodurans* R1 strains exposed to UV light. Exponential phase cultures of *D. radiodurans* R1, TNK101 *ΔddrC*, TNK102 *ΔddrB*, TNK103 *ΔddrD*, TNK104 *ΔddrA*, TNK105 *ΔpprA*, TNK106 *ΔrecA*, and LSU2000 *uvrA*1 *uvs*::Tn*DrCat* were exposed to UV light at 25 J m^-2^s^-1^. Values are the means ± standard deviations of three independent experiments (n = 9).

Strains TNK101 *ΔddrC*, TNK102 *ΔddrB*, TNK103 *ΔddrD*, and TNK104 *ΔddrA*, and TNK105 *ΔpprA* were evaluated for their ability to tolerate exposure to UV light (Figure 1) relative to their parent. Single deletions of *ddrA*, *ddrB*, *ddrC*, or *ddrD* have no effect on the UV resistance of *D. radiodurans* R1. Only deletion of *pprA* had a demonstrable effect on UV resistance. The reduction in the mean survival of TNK105 *ΔpprA* becomes statistically significant (unpaired t test, *p*=0.01, degrees of freedom (df) = 16) relative to R1 at 500 J/m^2^, at 1000J/m^2^ TNK105 is approximately eight-fold more sensitive to UV light when compared to R1.

For comparison, the survival curves for the UV sensitive strains LSU2000 [9] and TNK106 [16] are also included in Figure 1. LSU2000 carries a deletion in the *uvrA1* (DR1771) coding sequence and an insertion into the *uvs* (DR1819) gene [9], eliminating excision repair by the UvrABC complex and the species’ UV damage endonuclease (UVDE). TNK106 is a *ΔrecA* strain incapable of RecA-dependent homologous recombination [16].

### Deletion of *ddrA* (DR0423) in a *ΔpprA* background sensitizes *D. radiodurans* R1 to UV light

**Figure 2:**
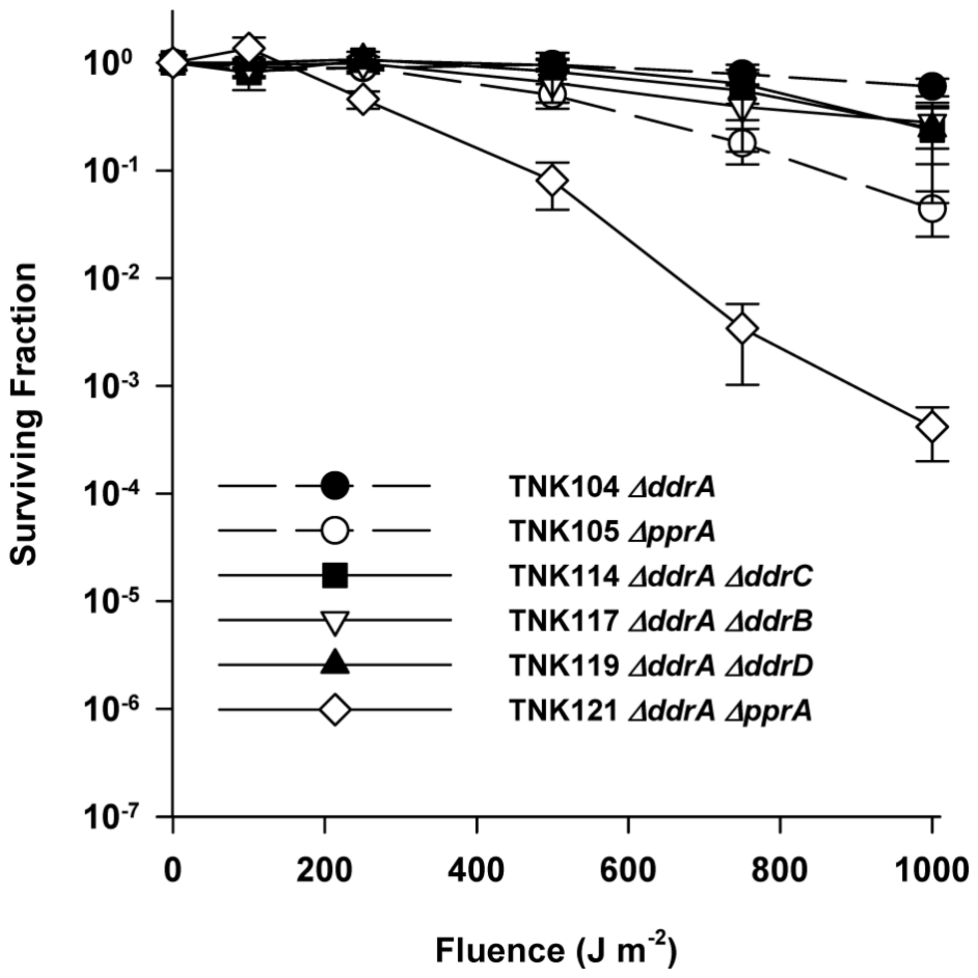
Survival curves for *ΔddrA* derivatives of *D. radiodurans* R1 exposed to UV light. The UV resistances of TNK114 *ΔddrA ΔddrC*, TNK117 *ΔddrA ΔddrB*, TNK119 *ΔddrA ΔddrD*, TNK121 *ΔddrA ΔpprA* are compared with TNK104 *ΔddrA*, TNK105 *ΔpprA*, and *D. radiodurans* R1. Values are the means ± standard deviations of four independent experiments (n = 12).

Deleting *ddrA* has no effect on the UV resistance of R1 (Figure 1), and strains TNK114 *ΔddrA ΔddrC*, TNK117 *ΔddrA ΔddrB*, and TNK119 *ΔddrA ΔddrD* are as resistant TNK104 *ΔddrA* (Figure 2). However, TNK121 *ΔddrA ΔpprA* demonstrates a rapid drop in UV resistance; there is a statistically significant (unpaired t test, *p*<0.0001, df = 22) two-fold reduction in the mean survival of TNK121 relative to TNK105 *ΔpprA* after exposure to a fluence of 250 Jm^-2^. TNK121 is 100-fold more UV sensitive than TNK105 *ΔpprA* (Figure 2) at 1000 Jm^-2^, indicating that DdrA functions in UV resistance in *D. radiodurans*, but its effect on survival is only apparent in the absence of a functional PprA.

### The combined deletion of *ddrB* (DR0070) and *ddrC* (DR0003) decreases the UV resistance of *D. radiodurans* R1

**Figure 3:**
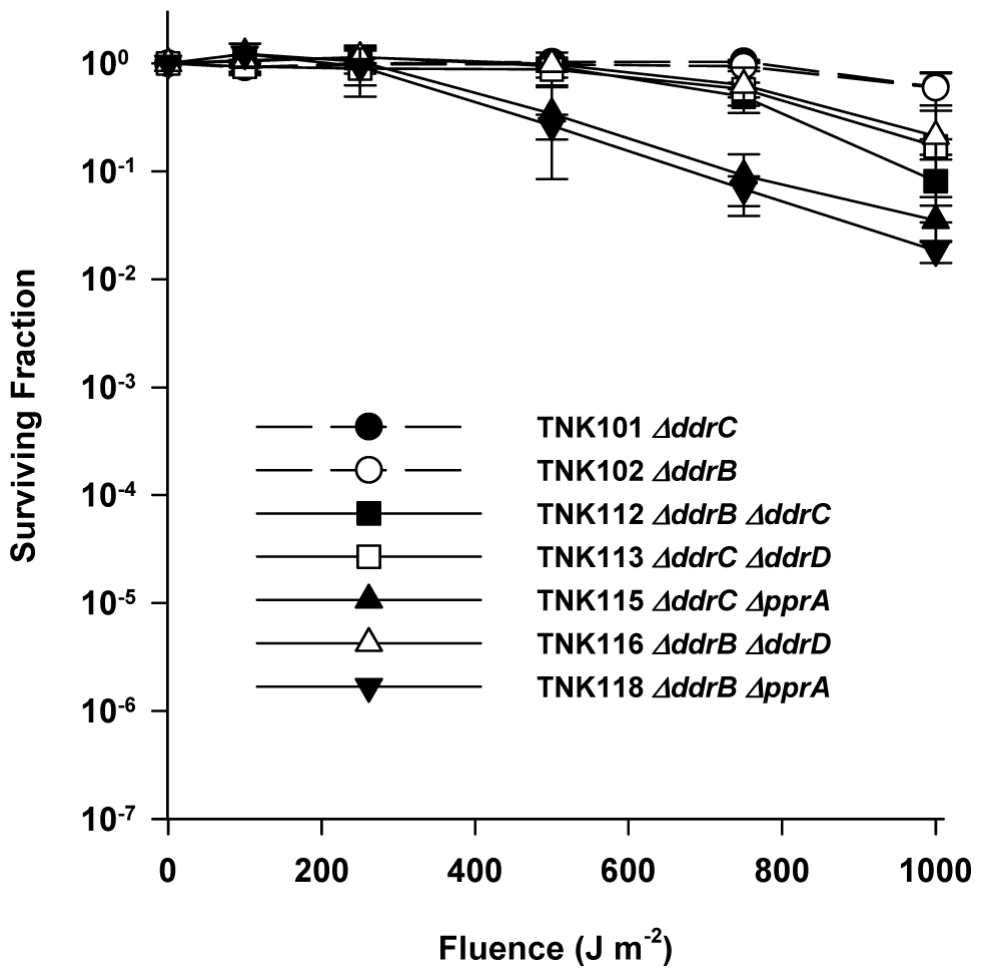
Survival curves for *ΔddrB and ΔddrC* derivatives of *D. radiodurans* R1 exposed to UV light. The UV resistances of TNK112 *ΔddrB ΔddrC*, TNK113 *ΔddrC ΔddrD*, TNK115 *ΔddrC ΔpprA*, TNK116 *ΔddrB ΔddrD*, and TNK118 *ΔddrB ΔpprA* are compared with TNK101 *ΔddrC*, and TNK102 *ΔddrB*. Values are the means ± standard deviations of six independent experiments (n = 18).

We find little evidence that DdrB or DdrC participate in UV resistance in *D. radiodurans*. TNK101 *ΔddrC* and TNK102 *ΔddrB* are as resistant to UV light as R1 (Figure 1). TNK113 *ΔddrC ΔddrD* and TNK116 *ΔddrD ΔddrB* do not significantly alter survival relative to TNK101, TNK102, or TNK103 *ΔddrD* (Figure 3). TNK115 *ΔddrC ΔpprA* is more sensitive to UV light relative to TNK101 *ΔddrC*, but its survival curve was identical to that of the UV sensitive strain TNK105 *ΔpprA* (Figure 1), indicating that loss of PprA alone was responsible for the sensitization observed.

The double mutant TNK112 *ΔddrB ΔddrC* is approximately ten-fold more sensitive than TNK101 or TNK102 (Figure 3) after exposure at 1000 Jm^-2^. Although this increased sensitivity is reproducible (n=18, six independent trials, three replicates per trial), it is the only evidence that DdrB or DdrC affect UV resistance at the exposures examined.

### Deletion of *ddrD* (DR0326) decreases the UV resistance of the *ΔpprA* strain TNK105

**Figure 4:**
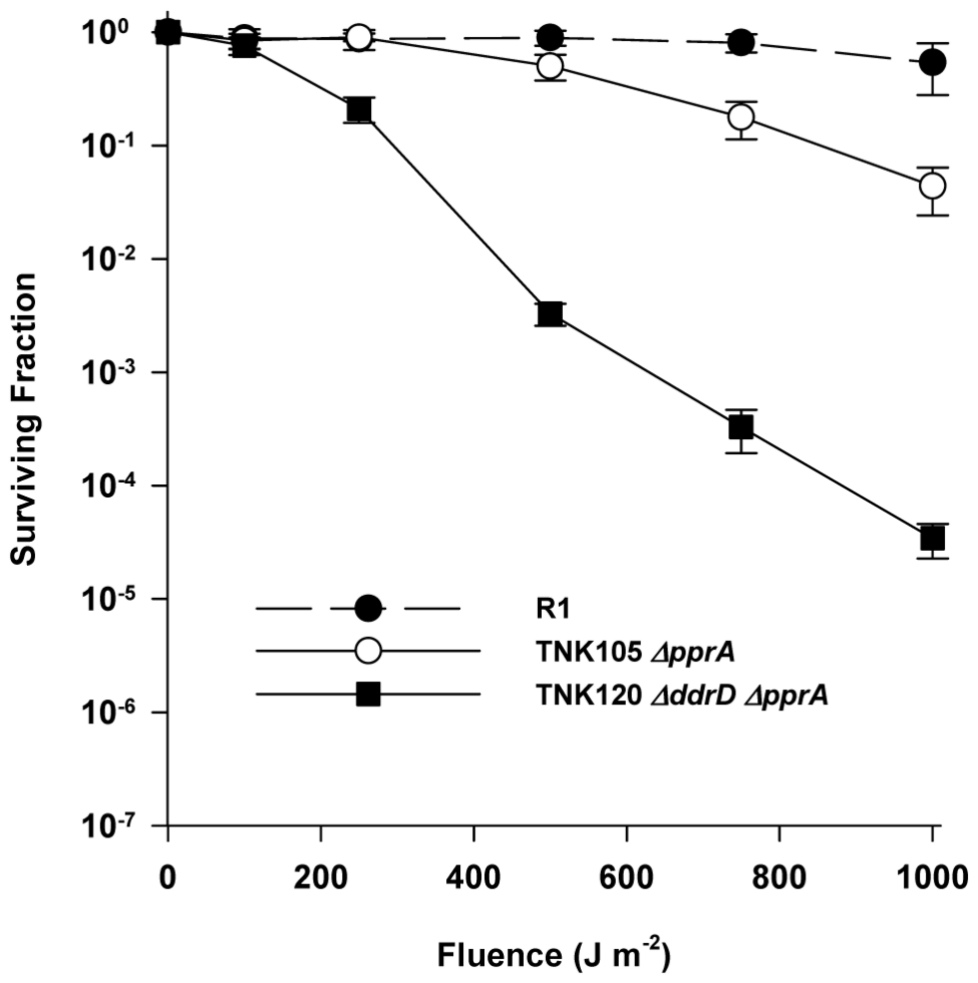
The survival curve of TNK120 exposed to UV light. The UV resistances of TNK120 *ΔddrD ΔpprA* compared with the survival curves of TNK103 *ΔddrD*, TNK104 *ΔddrA*, and TNK105 *ΔpprA*. Values are the means ± standard deviations of four independent experiments (n = 12).

Deletions of *ddrD* alone (Figure 1) or in combination with deletions of *ddrA* (Figure 2), *ddrB* (Figure 3), or *ddrC* (Figure 3) do not demonstrate sensitivity to UV light. However, the combination of *ΔddrD* and *ΔpprA* in TNK120 dramatically increases UV sensitivity relative to TNK105 *ΔpprA* (Figure 4). At 250 Jm^-2^, there is a significant (unpaired t test, *p*<0.0001, df = 25) ten-fold difference in viability and at 1000 J/m^-2^ TNK120 is three orders of magnitude more sensitive than TNK105.

### Deletion of *ddrA* (DR0423) sensitizes *D. radiodurans* R1 to mitomycin C

To further assess whether DdrA, DdrD, and PprA participate in excision repair or homologous recombination, strains carrying deletions of loci encoding these proteins were evaluated for resistance to the cross-linking agent mitomycin C (MC). Mitomycin C is highly toxic to prokaryotic cells [19]. After enzymatic reduction within the cell, MC generates reactive species that form DNA interstrand cross-links (ICLs) and a variety of guanine monoadducts in the minor groove [20,21]. There is an obligatory requirement for homologous recombination and NER during the repair of MC-induced DNA damage by *D. radiodurans* [22,23]. At their D_37_ dose, *uvrA* and *recA* defective strains are 60 and 300 times more sensitive to MC than R1, respectively.

**Figure 5:**
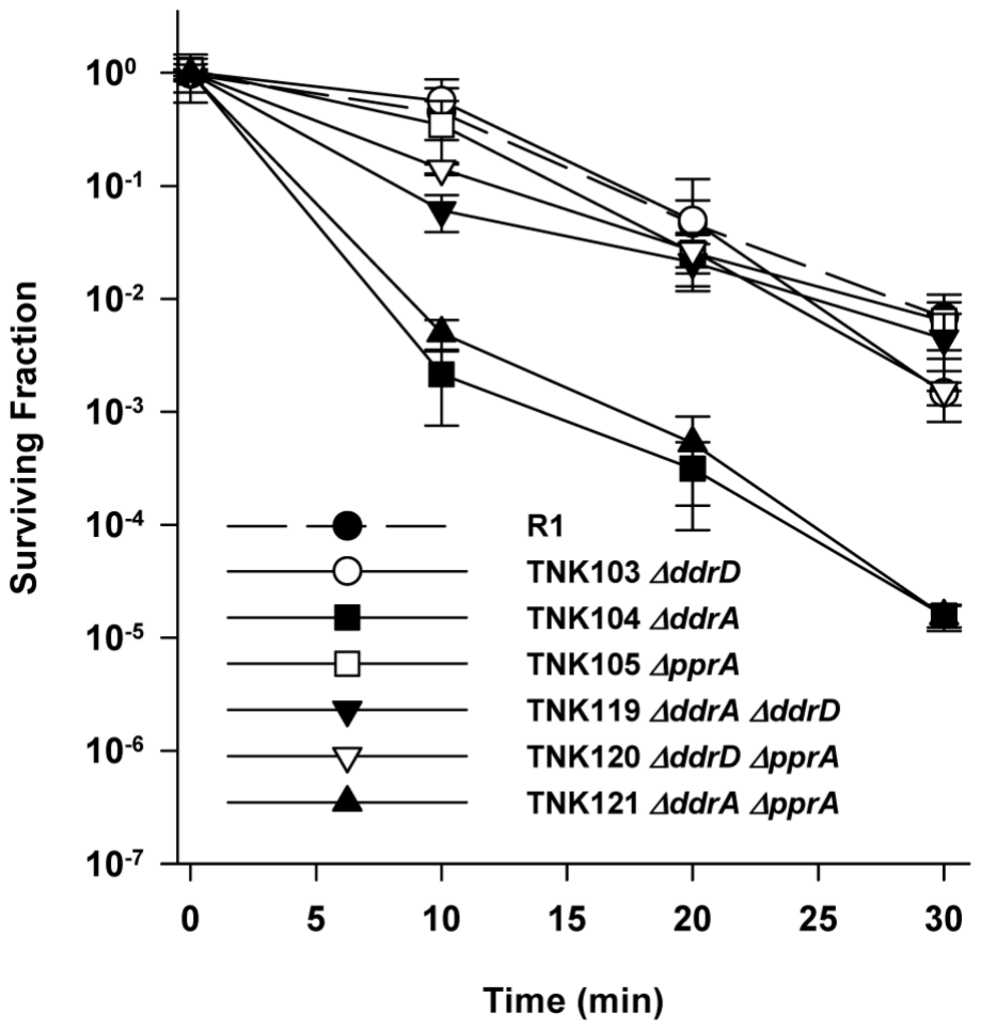
Survival curves for *ΔpprA* derivatives *D. radiodurans* R1 exposed to mitomycin C. TNK120 *ΔddrD ΔpprA* and TNK121 *ΔddrA ΔpprA* are compared with *D. radiodurans* R1, TNK103 *ΔddrD*, and TNK105 *ΔpprA*. Values are the means ± standard deviations of three independent experiments (n = 9).

TNK103 *ΔddrD* and TNK105 *ΔpprA* are as resistant to MC as R1 (Figure 5). In contrast, TNK104 *ΔddrA* cultures are very sensitive to mitomycin C (MC) relative to R1 (Figure 5) [24]; at each time interval sampled, R1 is between 150- and 400-fold more resistant to this reagent. Combining *ΔddrA* with *ΔpprA* does not change MC resistance; the sensitivity of TNK121 *ΔddrA ΔpprA* is indistinguishable from that of TNK104, indicating that loss of the DdrA is solely responsible for the phenotype. TNK120 *ΔddrD ΔpprA* is as resistant to MC as TNK103 *ΔddrD*, TNK105 *ΔpprA*, and R1 (Figure 5), suggesting that the sensitivity observed when *ddrD* and *pprA* are simultaneously deleted (Figure 4) is specific to UV-induced damage.

### Deletion of *ddrC* (DR0003) or *ddrD* (DR0326) restores mitomycin C resistance to a *ΔddrA* (DR0423) strain of *D. radiodurans* R1

**Figure 6:**
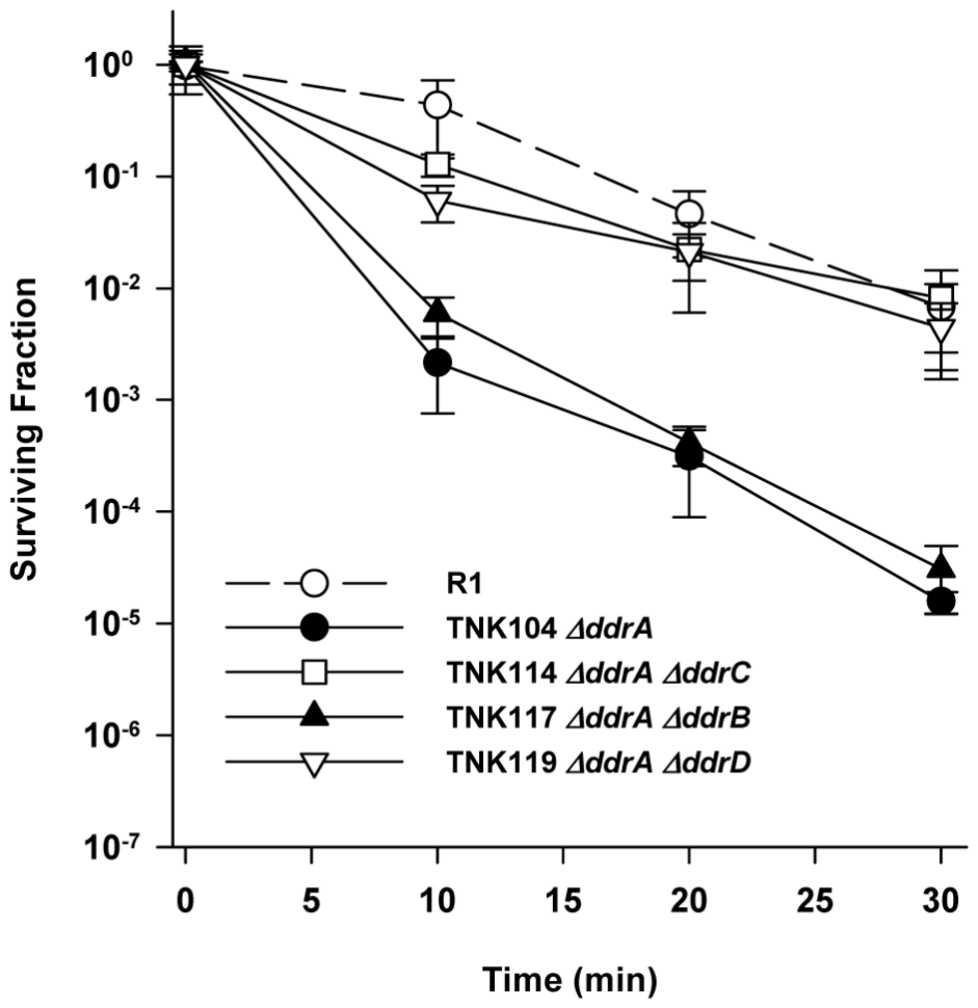
Survival curves for *ΔddrC and ΔddrD* derivatives of TNK104 *ΔddrA* exposed to mitomycin C. TNK114 *ΔddrA ΔddrC*, TNK117 *ΔddrA ΔddrB*, and TNK119 *ΔddrA ΔddrD* are compared with *D. radiodurans* R1 and TNK104 *ΔddrA*. Values are the means ± standard deviations of three independent experiments (n = 9).

**Figure 7:**
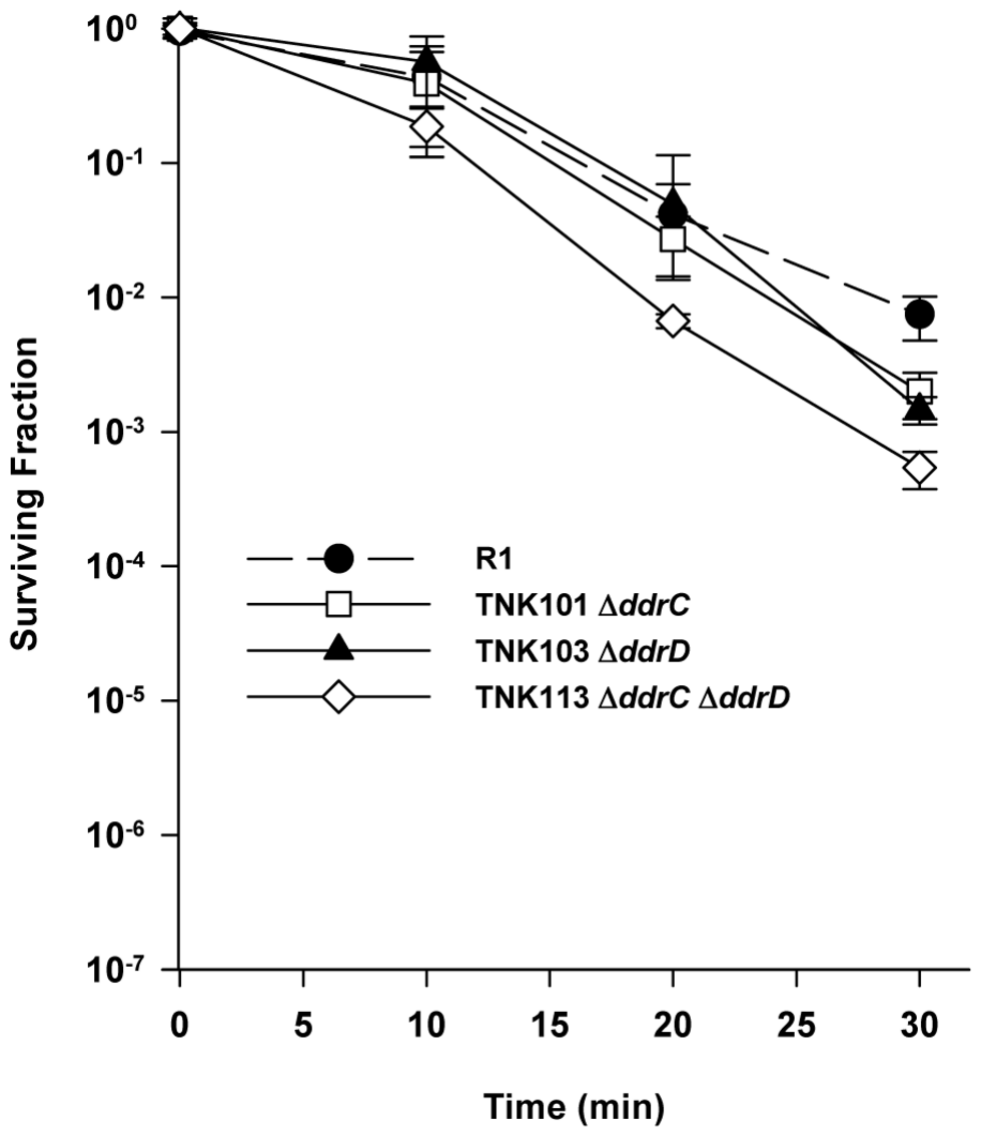
Survival curves for *ΔddrC* and *ΔddrD*, and *ΔpprA* derivatives *D. radiodurans* R1 exposed to mitomycin C. TNK101 *ΔddrC*, TNK103 *ΔddrD*, and TNK113 *ΔddrC ΔddrD* are compared with *D. radiodurans* R1. Values are the means ± standard deviations of eight independent experiments (n = 24).

Combining the *ddrA* deletion with deletions of either *ddrC* or *ddrD* restored wild type MC resistance to the resulting strains. TNK114 *ΔddrA ΔddrC* and TNK119 *ΔddrA ΔddrD* tolerate MC as well as R1 (Figure 6). TNK117 *ΔddrA ΔddrB* is as MC sensitive as TNK104 *ΔddrA*. TNK101 and TNK103 are no more sensitive to MC than is R1 (Figure 7). However, the double deletion TNK113*ΔddrC ΔddrD* exhibits slightly increased sensitivity to MC; there is a significant (unpaired t test, *p*<0.001, df = 45) 2.5-fold reduction in viability in TNK113 cultures relative to TNK101 and TNK103 at all time points evaluated.

### Deletion of *ddrC* (DR0003) or *ddrD* (DR0326) in a *ddrB* (DR0070) background significantly reduces the mitomycin resistance of *D. radiodurans* R1

**Figure 8:**
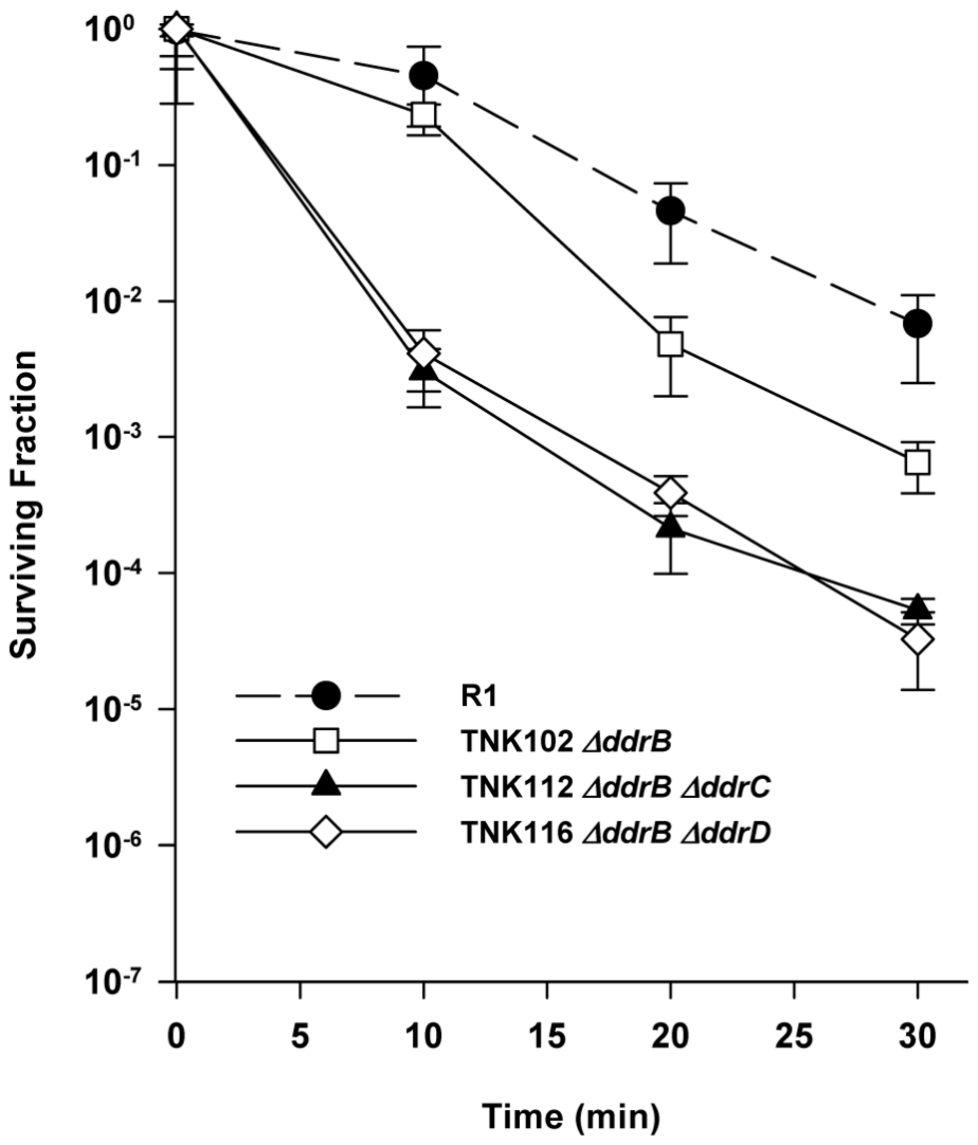
Survival curves for *ΔddrC* and *ΔddrD* derivatives *of* TNK104 *ΔddrB* exposed to mitomycin C. TNK102 *ΔddrB*, TNK112 *ΔddrB ΔddrC*, and TNK116 *ΔddrB ΔddrD* are compared with *D. radiodurans* R1. Values are the means ± standard deviations of eight independent experiments (n = 24).

As illustrated in Figure 8, TNK102 *ΔddrB* is approximately ten-fold more sensitive to mitomycin C relative to R1 at exposures of 20 minutes or longer. Including the *ΔddrC* or *ΔddrD* alleles in a *ΔddrB* background further reduces cell viability on exposure to this DNA damaging agent. TNK112 *ΔddrB ΔddrC* and TNK116 *ΔddrB ΔddrD* are between 100- and 150-fold more sensitive to MC than R1 at all exposure times.

## Discussion

*Deinococcus radiodurans* R1 is the type species for a family of bacteria characterized by its exceptional capacity to tolerate DNA damage [2,25,26]. Notably, exponential phase cultures of this species can withstand exposure to 5000Gy ionizing radiation and 500Jm^-2^ UV light; doses that will all but eradicate many bacterial cultures. Wild-type *D. radiodurans* cultures are also more resistant to ICLs than are most vegetative bacteria [27,28,29]. This species survives incubation in the presence of 20µg ml^-1^ MC for 10 min at 30^o^C with minimal loss of viability. Kitayama [28] reported that under these conditions greater than 90% of isolated genomic DNA exists as non-denaturable double stranded DNA with an average molecular weight of 2 x 10^7^ Daltons, and estimated that this level of exposure corresponds to approximately 100 MC-induced ICLs per *D. radiodurans* genome [28,29]. Approximately 20 ICLs are sufficient to inactivate repair proficient strains of *Escherichia coli* B/r [30].

In this study, we have demonstrated that the proteins DdrA, DdrD, and PprA play critical but previously unidentified roles in the UV resistance of *D. radiodurans* R1. The deletion of *pprA* in a wild type background sensitizes the resulting strain to UV light (Figure 1) and that sensitivity dramatically increases when *ddrA* or *ddrD* is also deleted from this background (Figures 2 and 4). The level of sensitivity we report for TNK120 *ΔddrD ΔpprA* is comparable to that observed in an *uvrA uvs* background that cannot carry out NER- and UVDE-mediated excision repair (Figures 1 and 4). TNK121 *ΔddrA ΔpprA* is approximately ten-fold more UV resistant than TNK120, but the strain remains significantly more sensitive than *D. radiodurans* R1 (Figure 2). The enhanced UV sensitivity of TNK120 and TNK121 suggest that the activities of PprA overlap with those of DdrA and DdrD; either they catalyze similar functions or they are required for separate processes that have an equivalent effect on UV resistance. The central role of homologous recombination and excision repair in reversing UV and mitomycin C-induced DNA damage in other species has been recognized for many years [31,32] forcing us to consider the possibility that DdrA, DdrD, and PprA contribute to these processes in *D. radiodurans*.

TNK103 *ΔddrD*, TNK104 *ΔddrA*, TNK105 *ΔpprA*, TNK119 *ΔddrA ΔddrD*, TNK120 *ΔddrD ΔpprA* and TNK121 *ΔddrA ΔpprA* are considered recombination-proficient [16]. These strains undergo natural transformation, a process that requires RecA-dependent recombination, with efficiencies identical to that of R1. In addition, the double mutants TNK119*ΔddrA ΔddrD*, TNK120 *ΔddrD ΔpprA* and TNK121 *ΔddrA ΔpprA* display varied responses to DNA damaging agents. TNK119 is modestly sensitive to IR [16], but as resistant as R1 to UV (Figure 2) and mitomycin C (Figure 6). TNK120 is sensitive to UV (Figure 4), but resistant to IR [16] and mitomycin (Figure 5). TNK121 is sensitive to IR [16], mitomycin (Figure 5), and UV (Figure 2). Since homologous recombination is required to survive exposure to IR, UV, and ICLs in *D. radiodurans* [22], the different patterns of resistance among these strains suggests that if DdrA, DdrD, or PprA contribute to DNA repair involving homologous recombination, their role is constrained in a manner specific to the DNA damaging agent or the lesions generated by that agent.

For similar reasons, it also seems unlikely that DdrA, DdrD or PprA are affecting excision repair. TNK120 is sensitive to UV and resistant to mitomycin. If one postulates that the inactivation of DdrD and PprA resulted in an excision repair defective strain, these opposing phenotypes cannot be reconciled. In addition, the UV sensitive double mutants TNK120 and TNK121 are wild type with respect to the *uvrA*, *uvrB*, *uvrC*, *uvs*, and *polA* genes that encode for excision repair systems capable of dealing with all major forms of UV-induced DNA damage [9]. It is difficult to comprehend how eliminating PprA and DdrA or PprA and DdrD could stop the contributions of NER and UVDE to UV resistance unless combined loss of these proteins influence UV resistance by affecting the function of both excision repair pathways simultaneously – in effect, recreating the situation that arises when the NER and UVDE systems are both inactivated. This scenario could occur if loss of DdrA and PprA or DdrD and PprA affected the stability of an intermediate or the activity of a component shared by the two pathways. At present, there is no evidence to support this idea.

The function of PprA is obscure; the protein has not been extensively characterized. Although homologues of PprA are encoded by all sequenced *Deinococcus* species [33,34,35], this protein shares no similarity to any other protein, amino acid sequence motif, or conserved domain described in the protein databases. Purified PprA is reported to stabilize the free ends formed at DNA double strand breaks and to recruit DNA ligase to the site of these breaks, improving the efficiency of ligation *in vitro* [36]. This report led to speculation that PprA performs the same function *in vivo*, and a suggestion that PprA participates in a form of non-homologous end joining (NHEJ) [37,38]. However, attempts to demonstrate NHEJ activity following *D. radiodurans* exposure to IR have failed to provide convincing evidence that this process is taking place [39,40]. While the results presented here do not directly support or refute the notion that *D. radiodurans* expresses the proteins needed for NHEJ, it is difficult to reconcile PprA’s central role in UV resistance with its possible involvement in NHEJ. Precedent suggests that NHEJ can play a minor role in repair of UV-induced DNA damage in bacteria [41]. The combined deletion of *ykoU* and *ykoV* of *Bacillus subtilis*, proteins that mediate NHEJ in this species, increases the strain’s sensitivity to UV light, but the effect is small, between one and a half and two-fold relative to the wild type at doses to 500 Jm^-2^. In contrast, the combined loss of PprA and DdrD results in a strain that is as much as 1000-fold more UV sensitive relative to the wild type (Figure 4). Whatever its activity, PprA makes a substantive contribution to UV resistance, and it seems doubtful that any contribution PprA may make to NHEJ fully defines its function following exposure to UV*.*

The ability of PprA to bind to the free ends created by DNA double strand breaks may explain why TNK121 *ΔddrA ΔpprA* is more sensitive to UV than TNK105 *ΔpprA* (Figure 2). DdrA is part of the Rad52 family of proteins [42]. In eukaryotes Rad52 promotes recombination and DNA double strand break repair through interaction with the Rad51 recombinase [43]. While DdrA contributes to the ionizing radiation resistance of *D. radiodurans* [16,24], there is no evidence that it behaves like the eukaryotic Rad52; the protein does not display DNA strand annealing activity [24]. In *D. radiodurans*, DdrA appears to function *in vitro* and *in vivo* by binding to the 3’ ends of single-stranded DNA and preventing DNA digestion by endogenous exonucleases following the cell’s exposure to high dose ionizing radiation [24]. It has been proposed that DdrA is part of a DNA end-protection system that helps to preserve genome integrity following DNA damage. In this capacity, it is not difficult to envision a situation where DdrA and PprA protect an intermediate created during repair of UV damage, and evidence of this protection is only obvious when both proteins are inactive.

The apparent overlap in DdrD and PprA activity is not as easily explained. Each protein appears to be a component of an independent process that facilitates UV resistance. DdrD function is unknown and this protein, which is found only in the *Deinococci* , shares no amino acid sequence similarity with any other characterized protein or sequence motif [33,34,35]. Inactivation of both proteins reveals their roles in UV resistance, but it is difficult to argue the DdrD acts to protect DNA as described for PprA and DdrA. PprA function is necessary if cells are to survive IR-induced damage, but DdrD seems to have only a minor role in IR resistance [16].

Figure 6 may provide a clue to DdrD function. TNK104 *ΔddrA* is two orders of magnitude more sensitive to mitomycin C than *D. radiodurans* R1. If deletions of *ddrC* or *ddrD* are inserted in this background, mitomycin resistance is restored to wild type levels. This result suggests that DdrC and DdrD are at least partially responsible for the increased sensitivity to mitomycin C observed in a *ddrA* strain. In other words, it appears that DdrA prevents a lethal event caused, directly or indirectly, by the wild type DdrC and DdrD proteins, and when either protein is inactivated that event is avoided. Like DdrD, DdrC shares no similarity with other proteins and is only known to be encoded by members of the genus *Deinococcus* .

Since inactivating DdrC and DdrD has the same effect in a *ΔddrA* background (Figure 6) or *ΔddrB* background (Figure 8), it is reasonable to ask if these proteins are part of the same repair complex. This possibility seems unlikely given the differences in UV sensitivity associated with TNK115 *ΔddrC ΔpprA* (Figure 3) and TNK120 *ΔddrD ΔpprA* (Figure 4); TNK120 is 1000 fold more UV sensitive when compared with TNK115. In addition, the simultaneous inactivation of *ddrC* and *ddrD* results in a strain with slightly decreased mitomycin C resistance (Figure 7). If DdrC and DdrD were different parts of a protein complex that carried out a single function, survival of the double mutant should not be different than TNK101 *ΔddrC* or TNK103 *ΔddrD*. Pending further investigation, we argue that the DdrC and DdrD proteins are components of different complementary processes involved in DNA damage tolerance.

At present, the phenotypes reported here are perhaps best explained by assuming that DdrA, DdrD, and PprA affect the efficiency of UV- and MC-induced lesion repair without directly catalyzing the removal of those lesions. We posit that these proteins suppress potentially lethal events that arise as repair proceeds following massive genetic insult, thereby facilitating survival. If this occurs, it may provide a context in which to better understand the extreme resistance of *D. radiodurans* to DNA damaging agents. We contend that these proteins assist the cell by allowing the cell enough time to achieve necessary repairs. Repair of damage after insult should be more effective if the time available for repair – the time between the appearance of damage and the lethal consequences of that damage – is extended. Assuming that a bacterial cell has the necessary repair proteins and sufficient time to carry out repairs, extreme tolerance to multiple DNA damaging agents could be conveniently explained.
